# Effects of electron beam, NaOH and urea on chemical composition, phenolic compounds, in situ ruminal degradability and in vitro gas production kinetics of pistachio by-products

**Published:** 2015-06-15

**Authors:** Masoud Moradi, Ahmad Afzalzadeh, Mehdi Behgar, Mohammad Ali Norouzian

**Affiliations:** 1 *Department of Animal Sciences, College of Abouraihan, University of Tehran, Tehran, Iran; *; 2 *Agricultural, Medical and Industrial Research School, Nuclear Science and Technology Research Institute, Karaj, Iran.*

**Keywords:** Pistachio by-product, Beam electron, Tannin, Gas production

## Abstract

The current study evaluated the effects of electron beam (EB; 10, 20, 30 and 40 kGy), urea and NaOH treatments on chemical composition, phenolic compounds, *in vitro* gas production and *in situ* ruminal degradability of pistachio by-product (PB). The EB decreased (*p* < 0.01) neutral detergent fiber (NDF), acid detergent fiber (ADF) and acid detergent lignin (ADL) contents of PB. Treating PB by NaOH and urea increased cell wall constitute of PB (*p* < 0.01). Irradiation at doses of 30 and 40 kGy reduced tannin and increased total phenol content of PB (*p* < 0.01). NaOH and urea treatments decreased tannins, total phenolics and condensed tannins of PB compared to control (*p* < 0.01). *In situ* ruminal dry matter (DM) degradation data showed that irradiation increased (*p* < 0.01) and, urea and NaOH decreased (*p* < 0.01) rapidly fermentable fraction (*a*) of PB. NaOH decreased (*p* < 0.01) fraction b and total degradable fraction (*a + b*) of PB. Irradiation and polyethylene-glycol increased rate of gas production (*k*), metabolizable energy (ME) and short chain fatty acid concentrations (SCFA), while, NaOH decreased gas production potential (*b*), ME and SCFA (*p* < 0.01). Data showed that although NaOH was efficient in detannification of PB, however, it reduced gas production and *in situ* degradation of PB.

## Introduction

Increasing of livestock population has resulted in shortage of feed sources and competition between humans and animals. These problems have led to increasing concern in using agricultural by-products in animal feeding. Iran is the first producer of pistachio in the world followed by USA and Turkey.^1^ High production of pistachio results in large quantity of pistachio by-products (PB). In Iran production of PB has been reported over 400,000 ton (based on fresh weight) annually. Main portions of PB consists of external shell (epicarp, 53.50%) followed by peduncles, leaves, mesocarp and kernel (27.70%, 9.50%, 5.30% and 4.00%, respectively) of the pistachio plant.^2^

Because of high level of crud protein (up to 16.00% of DM), ether extract (7.00% of DM) and NFC (40.00% of DM), and also moderate level of NDF (25.00% of DM), PB can be considered as a good feedstuff in ruminants diet.^3^ But this by-product contains high quantity of secondary metabolites like tannins ,7.50-9.50 % of total phenol and 3.50 to 4.50% of tannin; based on DM.^4^ Studies on lactating dairy cows and fattening lamb showed that PB cannot be included in their diets more than 15.0% and 30.0% (DM basis), respectively.^5^^,^^6^ For using large amount, phenolic compounds in PB must be diminished by appropriate treatments. Sodium hydroxide has been used successfully for detannification of tree and oak leaves.^7^^,^^8^ Urea has also been used for detannification of browses and oak leaves.^9^^,^^10^ More recently, ionizing radiations have been used by researchers in detannification of agricultural commodities.^11^ Behgar *et al*. reported gamma irradiation (at 10 kGy) of PB decreased 33.00% of tannin compared to un-irradiated group.^12^

The aim of this study was to determine effects of NaOH, urea and electron beam (EB) on chemical composition, phenolic compounds, *in situ* ruminal degradability and *in vitro* gas production parameters of PB.

## Material and Methods

**Pistachio byproduct preparation. **Pistachio by-products (Ohadi variety) were sampled from the different parts of a pistachio plantation (latitude 35°01’17”N, longitude 050°21’24”E), located 5 km Saveh, Iran, during the summer of 2011 and were mixed and sun dried before the experiment.

**Treatment application. **Pistachio by-products were packed in 14 polyethylene bags (15 × 20 cm). Eight bags were exposed to various doses (10, 20, 30 and 40 kGy, two bags per dose) of EB with a fixed beam energy of 10 MeV and dose rate of 180 kGy per min at room temperature by a Rhodotron accelerator (Model TT200; IBA Co., Louvain-la-Neuve, Belgium). Six other bags containing PB were also treated with 4% NaOH and 4% urea solution (40 g L^-1 ^of water) in the proportion of 1 L of each solution to 1 kg of samples (three bags for each treatment).^13^ Polyethylene bags were stored at room temperature under aerobic condition for three days.^13^

**Chemical composition. **Samples of treated PB were dried at 60 ˚C and afterwards milled to pass a 1 mm screen using a Wiley mill (Retsch Cutting Mill, Retschmule, Germany) and were analyzed for kjeldahl nitrogen (CP), organic matter (OM) and ether extract (EE) according to AOAC.^5^ Neutral detergent fiber (NDF), acid detergent fiber (NDF) and acid detergent lignin (ADL) was determined according to Van Soest *et al*.^14^ Total phenolics (TP), tannin (T) and condensed tannin (CT) contents were determined according to Makkar.^9^ All chemical analysis were done in triplicate.

**Nylon bag procedure. **Nylon bags (18.5 × 9 cm) with a pore size of 48 μm containing 5 g ground (2 mm screen) of treated PB (NaOH, urea and electron beam) were incubated in the rumen of three cannulated mature male Shaal sheep (triplicate samples) for 2, 4, 8, 16, 24, 48, 72 and 96 hr. Removed bags were washed under tap water until the rinse water was clear. Zero time disappears were obtained by washing un-incubated bags in a similar way. The percentage of DM disappearance at each time was calculated from the proportion remaining after incubation time relative to original feed.

***In vitro*** **gas production. **Gas production (GP) test was performed according to Menke *et al.*^15^ Approximately 200 mg of DM of each treatment was placed in triplicate 100 mL syringes and 30 mL of incubation medium was then added to them. Rumen fluid was collected from the three cannulated mature male Shaal sheep into a pre-warmed thermos flask, and then filtered and flushed with CO_2_. Gas production was measured at 2, 4, 6, 8, 12, 24, 48, 72 and 96 hr and a set of appropriate blanks (i.e., without PB) were included. Gas produced from each treatment was calculated by subtracting the produced gas form gas produced in the control blank. Effectiveness of treatments in detannification of PB was compared to polyethyleneglycol (PEG) as a reference treatment. In this regard, PEG (MW 6,000) treated PB (1:1) was prepared and used along with other treatments in gas production test. 

At the end of incubation (i.e., 96 hr), the pH of the content of each syringe was measured using a pH meter (Metrohm 691; Metrohm AG, Herisau, Switzerland).

Metabolizable energy (ME, MJ kg^-1^ DM) was estimated according to Menke *et al*. as:^15^

*ME = 2.20 + 0.13 GP (mL per 0.5 g DM) + 0.05 CP (g kg* ^-1^ *DM)*

Short chain fatty acid concentrations (SCFA) was calculated according to Menke *et al*. as:^15^

*SCFA (* *mmol* *per 200 mg DM) = 0.0222 GP *– *0.00425*

where, GP is the net gas production in 24 hr (mL per 200 mg of DM).

**Statistical analysis. **Gas production data were fitted using the non-linear option of SAS (Version 8.2; SAS Institute, Cary, USA) to the model as below: 

*G = b (1-e* ^-k(t)^ *)*

where, *G* is the volume of gas production at time *t*, *b* the asymptotic gas production (mL per 0.20 g DM) and *k* the rate of gas production (per hr) from the slowly fermentable feed fraction *b*. The data were of chemical composition and phenolic compounds analyzed by analysis of variance through the *F* test. The data of gas production were analyzed using the PROC MIXED for repeated measures by SAS. Tukey’s test was used to determine the differences amongst samples. Significant levels were defined as those with probabilities of 0.05 or less.

## Results

**Effects of treatments on chemical composition of PB. **The chemical compositions of experimental treatments are shown in Table 1. Urea treatment increased CP value of PB (24.45% for urea treated PB vs. 12.26% for control, *p* < 0.01) and NaOH decreased OM of PB (*p* < 0.01). Electron beam at 10-40 kGy decreased NDF, ADF, ADL and cellulose content of PB (*p* < 0.01). Addition of either urea or NaOH treatments increased (*p *< 0.01) the amounts of cell wall constituents (i.e. NDF, ADF and ADL) compared to control and irradiated groups. 

**Effects of tretmeants on the total phenolics, tannins and condensed tannins. **The effect of treatments on the total phenolic (TP), tannins (T) and condensed tannins (CT) are shown in Table 2. The EB decreased (*p *< 0.01) T content and increased (*p *< 0.01) TP content of PB. NaOH and urea decreased (*p *< 0.01) TP, T and CT of PB.

***In situ*** **ruminal degradability of treatments in sheep. **The effects of treatments on fraction *a*, fraction *b*, disappearance rate (*k*) and degradable fraction (*a+b*) of treated PB are shown in Table 3. The fraction *b* and degradable fraction did not differ among control and irradiated PBP. Application of NaOH on PB decreased (*p *< 0.01) fraction *a* and degradable fraction compared to control and irradiated PB. Electron irradiation increased (*p *< 0.01) fraction a compared to control and NaOH treated PB.

**Table 1:** Effects of experimental treatments (doses of electron irradiation, NaOH and urea) on the chemical composition of PB (% of DM).

**Treatment**	**Organic matter**	**Crude ptotein**	**Ether extract**	**NDF** 1	**ADF** 2	**ADL** 3	**Cellulose**
**Control**	88.19^a^	12.26^b^	6.81	25.93^c^	22c	8.56^c^	13.43^b^
**10 kGy**	88.23^a^	12.40^b^	6.92	24.14^d^	20.32^d^	7.56^d^	12.76^c^
**20 kGy**	88.11^a^	12.56^b^	6.94	24.28^d^	20.42^d^	7.15^d^	12.91^bc^
**30 kGy**	88.32^a^	12.53^b^	7.29	24.58^d^	20.46^d^	7.69^d^	12.78^c^
**40 kGy**	88.26^a^	12.49^b^	7.17	24.38^d^	19.70^e^	7.69^d^	12.01^d^
**Urea**	88.24^a^	24.45^a^	6.74	29.52^b^	22.43^b^	9.27^b^	13.16^cb^
**NaOH**	83.29^b^	12.39^b^	7.02	34.29^a^	34.19^a^	16.14^a^	18.05^a^
***p-*** **value**	< 0.01	< 0.01	> 0.01	< 0.01	< 0.01	< 0.01	< 0.01
**SEM**	0.14	0.12	0.13	0.09	0.08	0.13	0.11

1 Neutral detergent fiber,

2 acid detergent fiber, and

3 acid detergent lignin.

abcd Different superscripts in each column indicate significant differences (*p* < 0.01).

**Table 2:** Effects of treatments on the total phenolics, tannins and condensed tannins (% of DM) of pistachio by-product

**Treatments**	**Total phenolics**	**Tannins**	**Condensed tannins**
**Control**	10.92bcb	7.32^a^	4.36^a^
**10 kGy**	11.70^a^	7.61^a^	4.09^a^
**20 kGy**	11.65^a^	7.39^a^	4.96^a^
**30 kGy**	11.32^ab^	6.48^b^	4. 84^a^
**40 kGy**	11.61^a^	6.63^b^	4.98^a^
**Urea**	10.30^c^	5.09^c^	2.59^b^
**NaOH**	2.77^d^	1.01^d^	1.76^b^
***p*** **-value**	< 0.01	< 0.01	< 0.01
**SEM**	0.13	0.11	0.18

abcd Different superscripts in each column indicate significant differences (*p* < 0.01).

**Table 3:** The effect of treatments on *in situ* digestibility of treated pistachio by-product

**Parameters**	**Treatments**							***p-*** **value**	**SEM**
	**Control**	**10 kGy**	**20 kGy**	**30 kGy**	**40 kGy**	**Urea**	**NaOH**		
***Coefficients***									
***a***	0.46^b^	0.49^ab^	0.49^a^	0.50^a^	0.51^a^	0.42^c^	0.37 d	< 0.01	0.01
***b***	0.40^bc^	0.38^dc^	0.38^dc^	0.36^d^	0.34^d^	0.43^ab^	0.44^a^	< 0.01	0.01
***k***	0.054	0.05	0.05	0.05	0.06	0.07	0.04	< 0.01	0.01
**Degradable fraction (a+b)**	0.87 a	0.86 a	0.87^a^	0.86 a	0.85^a^	0.85^a^	0.81^b^	< 0.01	0.01

abcd Different superscripts in each column indicate significant differences (*p* < 0.01).

***In vitro*** **gas production parameters. **The effects of treatments on the cumulative gas produced at different times of incubation, fraction *b* and *k*, *in vitro* fermentation profile are shown in Table 4. Electron beam increased cumulative gas production in PB at all times of incubation compared to control (*p *< 0.01). However, this effect was significant only at 12 hr of incubation. Irradiation at 40 kGy increased gas production rate compared to other groups (*p *< 0.01). Addition of urea to PB decreased (*p *< 0.01) cumulative gas production at the 4 to 12 hr of incubation times compared to control. NaOH treatment decreased cumulative gas production at 6 to 96 hr of the incubation times compared to control (*p *< 0.01). NaOH decreased fraction *b* compared to other treatments (*p *< 0.01). Addition of PEG to PB increased volume of produced gas up to 72 hr of incubation and gas production rate compared to control (*p *< 0.01). Except for NaOH, other treatments increased ME of PB versus control (*p *< 0.01). While treatment with NaOH and urea decreased SCFA production (*p *< 0.01). 

**Table 4:** The effect of treatments on the volume of gas produced (cumulative) at different times, gas production parameters (*b* and *k*) and* in vitro* fermentation profile

**Parameters**		**Treatments**							***p-*** **value**		**SEM**
	**Control**	**10 kGy**	**20 kGy**	**30 kGy**	**40 kGy**	**Urea**	**NaOH**	**PEG**	**Treatment**	**Time**	
***Incubation time (hr)***											
**2**	5.09^bcd^	6.26^bc^	5.98^bc^	6.90^ab^	6.90^ab^	4.04^d^	4.9^cd^	8.12^a^	< 0.01	< 0.01	0.24
**4**	8.48^bc^	9.71^b^	9.13^b^	10.24^b^	10.27^b^	6.46^d^	7.07^cd^	12.19^a^	< 0.01	< 0.01	0.25
**6**	10.93^b^	12.50^ab^	11.69^b^	12.93^ab^	12.91^ab^	8.22^c^	8.19^c^	14.77^a^	< 0.01	< 0.01	0.31
**8**	13.10^b^	14.64^b^	14.02^b^	14.99^b^	14.99^b^	9.95^c^	9.22^c^	17.02^a^	< 0.01	< 0.01	0.26
**12**	16.12^c^	17.65^b^	17.06b^c^	18.10^b^	18.20^b^	13.02^d^	10.98^e^	20.09^a^	< 0.01	< 0.01	0.18
**24**	22.89^b^	24.76^ab^	24.20^b^	24.72^ab^	24.79^ab^	20.21^c^	17.09^d^	27.06^a^	< 0.01	< 0.01	0.34
**48**	28.71^ab^	30.99^a^	30.90^a^	31.28^a^	31.82^a^	26.67^b^	22.47^c^	31.85^a^	< 0.01	< 0.01	0.49
**72**	31.59^bc^	34.51^ab^	34.62^ab^	34.64^ab^	34.48^ab^	29.85^c^	26.48^d^	36.01^a^	< 0.01	< 0.01	0.48
**96**	33.65^ab^	36.38^a^	36.29^a^	35.83^a^	37.57^a^	31.47^b^	27.50^c^	37.37^a^	< 0.01	< 0.01	0.53
***Coefficients***											
***b***	32.38^ab^	34.80^a^	34.15^ab^	34.12^ab^	35.24^a^	30.93^b^	27.06^c^	34.40^ab^	< 0.01	< 0.01	0.48
***k***	0.055^cd^	0.061^bcd^	0.056^bcd^	0.063^bc^	0.070^ab^	0.053^d^	0.050^d^	0.083^a^	< 0.01	< 0.01	0.001
***In vitro fermentation profile***											
**pH**	6.63	6.61	6.58	6.57	6.56	6.62	6.60	6.58	> 0.01	< 0.01	0.03
**SCFA (mmol g** ^-1^ ** DM)**	0.52^a^	0.55^a^	0.53^a^	0.55^a^	0.56^a^	0.44^b^	0.37^c^	0.58^a^	< 0.01	< 0.01	0.02
**ME (MJ kg** ^-1^ ** DM)**	5.17^d^	5.30^c^	5.29^c^	5.31^c^	5.32^c^	6.68^a^	4.86^e^	5.41^b^	< 0.01	< 0.01	0.02

## Discussion

No data are available on the effects of ionizing radiation on chemical composition of PB. The effect of EB on OM, EE and CP in the present study was consistent with the previous studies in which no effects of EB were observed in chemical composition of whole cotton seed, sorghum grain and canola meal.^16^^-^^18^ Similarly, no effect of EB was observed on the chemical compositions of gamma irradiated soybean meal up to 75 kGy.^19^

Increase in CP of PB in the urea treatment could be due to the high amount of nitrogen in urea molecule, while decrease in OM matter of NaOH treated PB could be explained by the presence of Na molecules in this treatment. Similarly, Arisoy showed an increase in ash content for NaOH treated straw.^6^

 The decrease in cell wall of the PB with increasing irradiation dose is in contrast to the results of the previous studies in which ionizing radiation of agricultural by-product did not show any change in cell wall constituent.^17^^-^^18^ However, in agreement with our finding, some studies showed decrease in cell wall constituent (NDF, ADF and ADL) of agricultural by-products using high dose of gamma irradiation.^20^^-^^21^

In the present study, the decrease in NDF and ADF was caused by the effect of irradiation on ADL and cellulose content of PB. These effects could explain the decrease of cell wall constitute (NDF and ADF) of the PB. All doses of irradiation (10 to 40 kGy) decreased NDF and ADL content of PB to the same extent compared to control, however, irradiation at 40 kGy decreased ADF and cellulose much more than other doses (19.70% and 12.01% for ADF and cellulose, respectively. 

Irradiation has been suggested to decrease polysaccharides (e.g., cellulose) mainly via cleavage of the glycosidic bonds.^22^ This leads to the formation of lower molecular weight sugars such as glucose and maltose.

Chemical treatments increased cell wall constituent of PB. This effect was more prononeced in the case of NaOH compared to urea treatment. These results are incontrast to the results of previous studies using other sources of agricultural by-products.^20^^,^^23^^-^^24^ Haddad *et al.* showed decrease in NDF content of NaOH treated (3.00% and 5.00%) wheat straw, however, no changes were observed in ADL and ADF content.^24^

Al-Masri and Guenther showed treatment of some agricultural by-products with 5% urea decreased the cell-wall constituents (NDF, ADF and ADL) over the control.^20^ Similarly, Banchorndhevakul reported a decrease in NDF of rice straw and corn stalk urea treated (5.00%) after storage at room temperature for 21 days.^23^

However, similar to the results in this study Arisoy showed that NaOH treatment of straw reduced the hemicellulose content and this resulted in an increase in the content of cellulose and lignin in the remaining cell wall.^6^

To the best khowledge of the authors there are no data available on the effects of chemical treatments on chemical composition of PB. In the present study the averag of tannin reduction of EB irradiated PB at 30 and 40 kGy was 10.50% compared to the control. Tannin reduction of PB by EB in the present study was consistent with result of Behgar *et al*. who reported gamma irradiation decreased T content of PB by 33.00% when PB were irradiated at 10 kGy.^25^

The level of TP was increased by the exposure of PB to the EB. In most of the reports using ionizing irradiation a decrease in the level of T was accompanied with an increase in TP.^26^^-^^28^ The increase in TP in the current study is in agreement with the result of Behgar *et al*. who reported numerically increase in TP content of gamma-ray treated PB.^25^

The effect of ionizing radiation on phenolic compounds increscent might be due to release of bound or insoluble phenolics from the broken cell wall components. No effect of irradiation was observed in CT content of PB in this study. 

The decrease in T content by NaOH and urea addition in the present study was in agreement with previous work.^29^ Ben Salem *et al*. found that low level of urea (20 g kg^-1^ fresh *Acacia*) treatment after seven days was sufficient to inactivate extractable TP, T and CT of *Acacia cyanophylla* leaves.^30^

Although there are no available data for the effect of NaOH and urea treatment on T content of PB, in one study the effect of ensiled PB with the addition of urea (0.15% of DM) showed approximately 19.00% and 30.00% reduction in TP and T, respectively.^31^ Among treatments the major decrease of T and CT in PB was occurred when BP was treated by NaOH. NaOH treatment caused approximately 74.63%, 86.20% and 59.63% reductions in TP, T and CT compared to control, respectively. 

The value of fraction *a* (0.46), *b* (0.40), and *k* (0.054 per hr) of DM degradability in untreated PB are within the range reported in the previous studies. ^31^^-^^32^ The irradiation increased fraction *a* of PB compared to control and chemical treatments that this effect was consistent with the effect of irradiation on cell wall of PB. The same result was noted when irradiated (100 to 300 kGy) wheat straw were incubated in the rumen of steers.^33^ This effect could be simply contributed to the degradation of cell wall polysaccharides to lower molecular weight sugars by radiation.

No effects of irradiation were noted on the total DM degradable portion (*a* + *b*). No effect of gamma radiation on DM degradable portion of canola meal also reported by Taghinejad *et al*.^18^ Generally its accepted that application of higher doses of ionizing irradiation (> 100 kGy) increase degradable portion of agricultural by-products in the rumen. Shahbazi *et al*. showed that electron irradiation (100 to 300 kGy) of wheat straw resulted in increase of rumen degradable portion compared to control.^34^ It has also been reported that gamma irradiation of barley straw and electron irradiation of alfalfa hay (both at 100 to 300 kGy) resulted in increase of rumen degradability of DM, NDF and ADF.^33^^-^^34^

Application of urea and NaOH on the PB decreased fraction “a” and degradable portion. This result was in line with effect of urea and NaOH on the cell wall content of PB.

To the best knowledge of the authors there is no data available on the effect of EB and chemical treatments on gas production parameter and *in vitro* digestion of PB. Behgar *et al*. showed no effect of gamma irradiation on comulative gas production of PB.^25^ Al-Masri and Zarkawi showed increase *in vitro* apparent organic matter digestibility and digestible energy of some agricultural by-products at various doses of gamma irradiation (5 to 150 kGy).^35^

In the present study in general chemical treatments decreased gas production. Canbolat *et al*. showed that NaOH treatment of *Arbutus andrachne* and *Glycyrrhiza glabra* leaves decreased *c* and *b* fractions of gas test.^29^

Kamalak *et al*. showed that fraction *b* in gas test was negativley correlated with total and soluble condenced tannin.^36^ In contrast to this finding, removal of CT by NaOH and urea treatments in the present study resulted in decrease gas volume and gas parameters. The decrease in fraction *b* was more pronounced for NaOH compared to urea treatment. The possible explanation of the effect of NaOH on reduced gas volume might be due to increase of cell wall constitute of PB in this treatment.

Treatment with PEG has been used in many nutritional *in vitro* and *in vivo* studies to negate adverse effects of tannins on the nutrient utilization by gut microbes and animals. Addition of PEG to tannin containing material led to increase in *in vitro* gas production.^37^ In the present study, addition of PEG increased gas production and gas production parameters. This result agrees with the study of Bagheripour *et al*. who found the addition of PEG increased gas volume of PB.^38^ Increase in gas production also reported by Getachew *et al*. in PEG treated tannin-containing browses.^37^ Also, PEG increased rate of gas production from 0.05 to 0.08. Similarly, numerical increase in gas production rate was noted in previous study in which PB were irradiated by gamma radiation at the doses of 10 to 60 kGy.^3^ Elevation of *in vitro* gas production of irradiated PB could be due to both detanninfication and cell wall reduction in EB treated PB.

Addition of PEG resulted in increased ME value (5.41 MJ kg^-1^ DM), which agrees with Bagheripour *et al*. who reported that PEG increased the ME content of PB.^38^ The same results were also noted by Yousef Elahi where PEG addition to the oak’s leaves increased ME in gas production.^39^ Difference in ME among treatments reflects variation in fermentable carbohydrates and available nitrogen among them.^40^ In the present study, increase of ME in EB treated PB at 10 to 40 kGy could be attributed simply to decrease in cell wall content and consequent increase in soluble sugar, while increase of ME in urea treated PB (6.68 MJ kg^-1^ DM) could be due to high nitrogen content of this treatment. Nonetheless, except for NaOH treated PB which had a low ME of 4.86 MJ kg^-1^, all other treatments can be considered as good source of ME. 

In the present study, urea and NaOH addition to PB decreased (0.44 and 0.37 mmol g^-1^ DM, respectively) SCFA compared to other treatments and control. This finding could be simply contributed to the decrease of produced gas due the increase of cell wall in these treatments as mentioned earlier. The gas production data in the present study supported *in situ* data.

In conclusion, improvements in ME, as well as rate of gas production with EB and PEG support their neutralizing effect on tannins. Although NaOH decreased tannin content of PB more effectively than other treatments, however, it resulted in diminish production of gas, ME, SCFA and *in situ* degradation of PB. Further study is needed to test the effectiveness and digestion of irradiated and chemical treated PB to be tested in* in vivo *trials.
